# Intertwin umbilical vein diameter-to-abdominal circumference discordance in type 1 selective fetal growth restriction twins: association with risk of perinatal death in the smaller twin

**DOI:** 10.3389/fmed.2026.1865572

**Published:** 2026-09-25

**Authors:** Minyu He, Chunyan Zhong, Jinwei Zhang, Peng Tu, Suzhen Ran, Jianjun Zheng, Jing Tang

**Affiliations:** 1 Department of Ultrasound, Chongqing Health Center for Women and Children, Chongqing, China; 2 Department of Ultrasound, Women and Children’s Hospital of Chongqing Medical University, Chongqing, China; 3 NHC Key Laboratory of Birth Defects and Reproductive Health, Chongqing, China; 4 Chongqing Key Laboratory of Human Embryo Engineering and Precision Medicine, Chongqing, China; 5 Department of Ultrasound, Youyang Tujia and Miao Autonomous County Liangzeng Central Health Center, Chongqing, China

**Keywords:** doppler, perinatal death, selective fetal growth restriction, twin pregnancy, umbilical vein

## Abstract

**Objective:**

Selective fetal growth restriction (sFGR) is a common complication of monochorionic-diamniotic twin pregnancies, and there is no unified clinical standard for determining the optimal timing of intervention. This study aimed to explore the value of ultrasound parameters for identifying risk of perinatal death (PND) of the smaller twin complicated with type 1 sFGR.

**Methods:**

This single-center retrospective cohort study included 49 women with type 1 sFGR twin pregnancies who received regular prenatal ultrasound examination and had complete clinical and follow-up data. Cases with type 2/3 sFGR, fetal structural or chromosomal abnormalities, severe maternal complications, intrauterine intervention, or loss to follow-up were excluded. The primary outcome was PND of the smaller twin with type 1 sFGR, defined as either intrauterine death or 28-day neonatal death. The patients were divided into the PND group (*n* = 19) and the non-PND group (*n* = 30). Baseline ultrasonic indices were compared, with nested logistic regression, reclassification analysis and 500-sample bootstrap internal validation performed to assess incremental value and model stability.

**Results:**

Gestational age (GA) at diagnosis and the intra-abdominal umbilical vein diameter to abdominal circumference ratio (UV/AC) of the smaller twin in the PND group were significantly lower than those in the non-PND group (*P* < 0.05), whereas intertwin UV/AC discordance was significantly higher (P0.05). Multivariate logistic regression identified two factors independently associated with PND in type 1 sFGR smaller twin: earlier GA at diagnosis (*P* = 0.013) and elevated intertwin UV/AC discordance (*P* = 0.006). The optimal cut-off of intertwin UV/AC discordance was 0.273% (AUC = 0.814, sensitivity 89.5%, specificity 53.3%). Reclassification analyses confirmed the incremental prognostic value of intertwin UV/AC discordance beyond conventional Doppler and clinical variables. The cutoff for GA at diagnosis yielded an AUC < 0.5, indicating reversed predictive performance.

**Conclusion:**

GA at diagnosis and intertwin UV/AC discordance are independently associated with PND in type 1 sFGR smaller twin. Earlier type 1 sFGR diagnosis and higher intertwin UV/AC discordance are correlated with an increased PND risk. Intertwin UV/AC discordance may serve as a promising ultrasonic marker for antenatal risk stratification in type 1 sFGR.

## Introduction

Selective fetal growth restriction (sFGR) is a rare but high-risk complication of monochorionic diamniotic (MCDA) twin pregnancies, occurring in 10%–15% of such gestations and associated with increased perinatal morbidity and mortality (1). Its adverse outcomes include intrauterine fetal demise, neonatal respiratory distress syndrome, and long-term neurodevelopmental disorders (2–5). Since sFGR was proposed in the 1980s, unequal placental sharing has been acknowledged as its core pathogenesis (6). Standardized diagnostic frameworks have been established by the 2016 ISUOG guidelines (7) and the 2019 international expert consensus, which defined key diagnostic indicators including GA (gestational age), umbilical and middle cerebral artery pulsatility indices, and inter-twin weight discordance (1). However, the optimal intervention timing and management strategies for sFGR remain controversial.

According to umbilical artery Doppler waveform classification, type 2 and type 3 sFGR are characterized by obvious fetal hemodynamic abnormalities, for which active intrauterine intervention or early pregnancy termination is usually recommended. In comparison, type 1 sFGR accounts for the majority of clinical cases and presents persistent normal umbilical artery diastolic flow during prenatal follow-up. The absence of typical arterial Doppler abnormalities renders conventional ultrasound indicators ineffective for early risk prediction, resulting in a lack of simple and reliable prognostic tools for clinical risk stratification of type 1 sFGR.

Prenatal ultrasound is the fundamental surveillance modality for sFGR, yet existing studies have notable limitations. Most previous studies focus primarily on fetal weight and umbilical artery parameters for routine monitoring rather than systematic prognostic assessment (8–10). Although inter-twin estimated fetal weight (EFW) discordance (≥25%) and cerebral hemodynamic changes have been linked to perinatal prognosis (11–16), most analyses enroll mixed sFGR subtypes without targeted verification in type 1 cohorts. In recent years, emerging evidence has demonstrated that umbilical vein(UV) parameters are valuable for predicting subclinical placental dysfunction and adverse fetal growth outcomes in growth-restricted singleton fetuses， even in cases with normal umbilical artery Doppler waveforms (17, 18). However, relevant investigations focusing on intertwin UV discrepancies and their prognostic performance in sFGR remain scarce, and the clinical application of UV-related indicators for type 1 sFGR risk stratification is still insufficiently explored.

The umbilical vein diameter to abdominal circumference ratio (UV/AC) has unique physiological and clinical strengths for solving this problem. The intra-abdominal UV diameter reflects fetal placental venous perfusion, while normalization by abdominal circumference eliminates individual fetal size interference. Compared with arterial indices that only reflect peripheral perfusion, intertwin UV/AC discordance can comprehensively evaluate placental hemodynamic imbalance and identify early hypoperfusion before arterial Doppler deterioration. Moreover, UV/AC measurement is convenient and fully compatible with routine prenatal ultrasound, which benefits early risk stratification in type 1 sFGR. Nevertheless, its prognostic value for adverse perinatal outcomes in pure type 1 sFGR remains unvalidated.

Given the above research gaps, this study aimed to explore the risk-stratification performance of conventional ultrasonic indicators and intertwin UV/AC discordance for perinatal death (PND) among type 1 sFGR smaller twin. We hypothesized that intertwin UV/AC discordance would be independently associated with PND in type 1 sFGR, and would show better discriminatory capacity compared with traditional Doppler and EFW discordance indicators.

## Methods

This is a retrospective study based on a single center. This study was approved by the Ethics Committee of Women and Children's Hospital of Chongqing Medical University/Chongqing Health Center for Women and Children (Registration No. (2024)-075).

### Case selection

The clinical data of 49 twin pregnancies diagnosed with type 1 sFGR via prenatal ultrasonography in our hospital from January 2018 to December 2025 were analyzed retrospectively. The inclusion criteria were as follows: (1) MCDA twin pregnancies confirmed with Type 1 sFGR by ultrasonography: the EFW of one twin is less than the 10th percentile for the corresponding GA, the intertwin EFW discordance is ≥25%, and the umbilical artery diastolic flow waveform of the smaller fetus is normal. (2) Pregnant women received standardized regular prenatal care throughout pregnancy. (3) Complete and retrievable medical charts, ultrasound reports, delivery records and neonatal follow-up data are available for MCDA twin gestations. Exclusion Criteria: (1) Cases complicated with Type 2 or Type 3 sFGR. (2) Fetuses with structural malformations, chromosomal abnormalities or inherited disorders. (3) Mothers suffering from severe maternal complications that could directly result in adverse fetal outcomes, such as severe preeclampsia, poorly controlled gestational diabetes mellitus, and severe pre-existing cardiac, hepatic or renal diseases. (4) Subjects lost to follow-up. (5) Patients who underwent any intrauterine interventional treatment.

### Ultrasonography and outcome

Pregnant women were placed in a supine position and routine ultrasound examination was performed to rule out fetal developmental abnormalities. Ultrasonography was performed with a GE Voluson E8 and E10 color ultrasound machine ((GE Healthcare Ultrasound, Milwaukee, WI, USA) with a frequency of 2 to 9 MHz convex array probe.

The fetal growth index was measured, including biparietal diameter, head circumference, abdominal circumference, and femoral length. EFW was calculated using the Hadlock formula, and the intertwin EFW discordance was determined as △EFW = ([EFW _larger twin_ –EFW _smaller twin_]/ EFW _larger twin_) × 100%). The ultrasonic Doppler parameters of the fetal umbilical artery (UA) and middle cerebral artery (MCA) were measured. UA was measured at the free-floating end of the umbilical cord, and MCA from the cross-section of the fetal head of the Willis ring. The umbilical artery S/D (UA-S/D), umbilical artery pulsatility index (UA-PI), middle cerebral artery-peak systolic velocity (MCA-PSV), and middle cerebral artery pulsatility index (MCA-PI) were measured. The cerebroplacental ratio (CPR) was calculated by dividing MCA-PI by UA-PI. The maximum diameter of the intra-abdominal umbilical vein (UV) was measured on the fetal abdominal transverse plane at the straight intra-abdominal segment, with slightly tilted to obtain a true cross-section of the vessel under grayscale B-mode imaging without color Doppler overlay (Figure 1). Calipers were placed on the inner-to-inner borders of the venous walls, and the image was magnified to optimize visualization of the vessel lumen. Three consecutive measurements of UV diameter were performed, and the average value was adopted for analysis. Abdominal circumference was measured following ISUOG guidelines. The UV/AC ratio for each twin was calculated as intra-abdominal UV diameter (mm) divided by abdominal circumference (mm). Intertwin UV/AC discordance was defined as (UV/AC _larger twin_–UV/AC _smaller twin_) × 100%, expressed as a percentage (%). Each twin was labeled and measured independently to avoid sampling bias.

**Figure 1:**
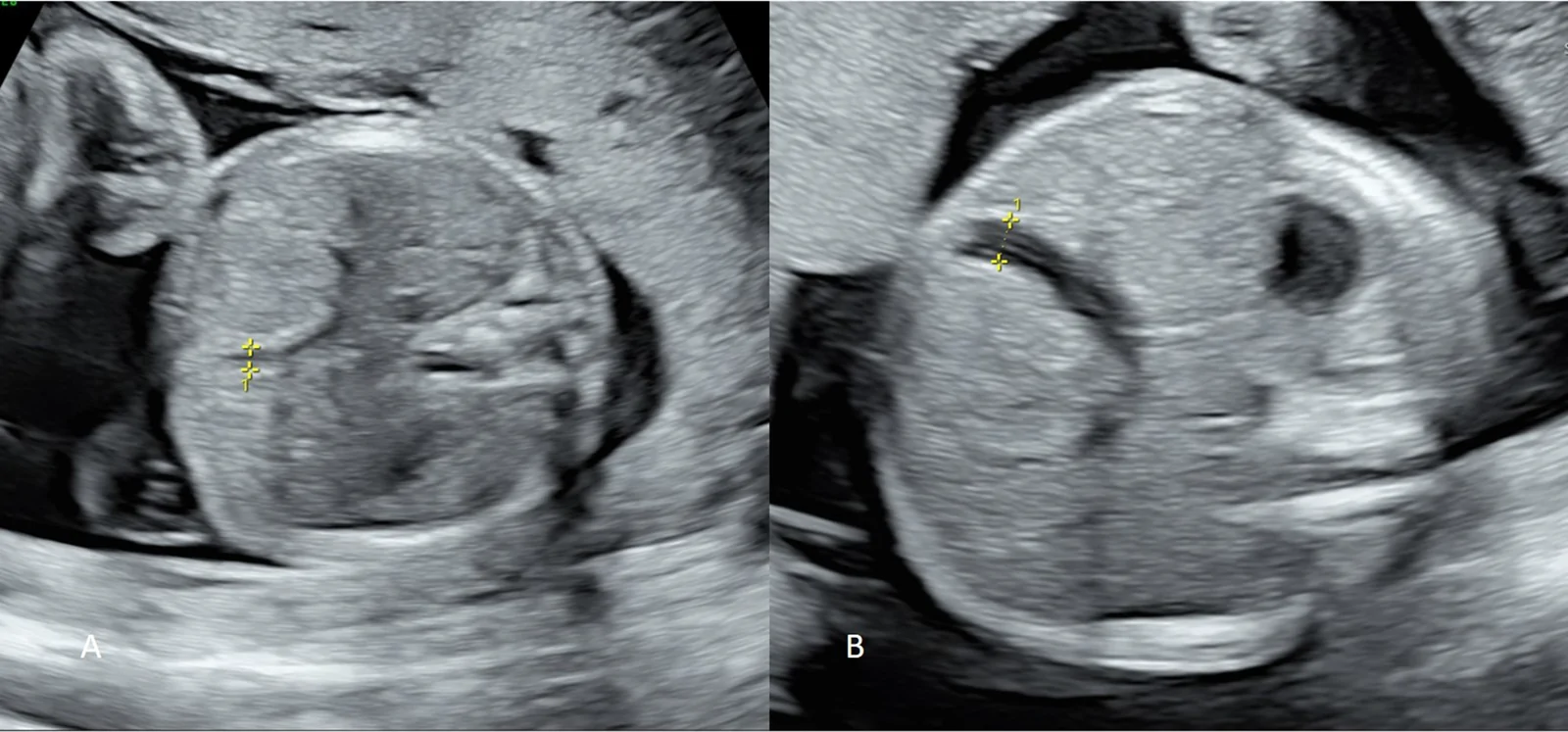
Ultrasonographic measurement of the intra-abdominal umbilical vein (UV)diameter on the fetal abdominal transverse plane in monochorionic diamniotic twin pregnancies complicated by selective fetal growth restriction (sFGR). **(A)** UV diameter measurement of the smaller twin; **(B)** UV diameter measurement of the larger twin.

Data were obtained from the ultrasonic results at the time of type 1 sFGR diagnosis. All ultrasound examinations were performed by two experienced fetal medicine sonographers. The pregnancy program was handled by the attending obstetrician, who did not participate in the ultrasound diagnosis process and was blinded to most data except UA-S/D and △EFW.

The primary outcome was PND of the smaller twin with type 1 sFGR, defined as either intrauterine death or neonatal death within 28 days after delivery. A total of 49 cases were enrolled in the present study, including 19 cases in the PND group and 30 cases in the control group (non-PND) without PND.

### Statistical analysis

SPSS Statistics 26 software and R software were used for statistical analysis. Data are expressed as a constituent ratio, and the comparison between groups was analyzed by *χ*^2^ test or the Fisher exact probability method. Data with normal distribution are expressed as mean ± standard deviation, and the student's t-test was used to compare two groups. Data with non-normal distribution are expressed by median (quartile spacing); the rank sum test was used for comparison between the two groups. Correlation analysis was performed using Pearson correlation analysis. Multivariate logistic regression analysis was used to explore the correlation strength between ultrasound parameters and PND. For stable model estimation, Z-score standardization was performed for the variable (intertwin UV/AC discordance) prior to regression to eliminate dimensional differences. Nested logistic regression models were constructed: the base model (Mod A) included conventional clinical and Doppler parameters, while the full model (Mod B) incorporated intertwin UV/AC discordance on the basis of Mod A. 500-times bootstrap resampling was applied for BCa bias-correction of odds ratios and for internal validation to assess model optimism and potential overfitting. ROC curves were plotted, and DeLong test was used for AUC comparison between nested models; NRI and IDI were calculated to evaluate incremental predictive value, with DCA adopted to assess clinical net benefit. ICC was used to assess intra- and inter-observer reproducibility of intra-abdominal UV diameter measurement. A two-sided *P* < 0.05 was considered statistically significant.

## Results

### Study population

A total of 49 pregnant women with type1 sFGR twins who underwent prenatal ultrasound examination in our hospital from January 2018 to December 2025 were retrospectively analyzed.

The maximum follow-up duration was 2 years after birth. A total of 49 cases were enrolled in this study. Among them, 19 cases were assigned to the PND group: 18 cases involved intrauterine demise of the smaller twin (12 cases of simultaneous dual-twin demise and 6 cases of isolated demise of the smaller twin), and 1 case experienced immediate postnatal death of the smaller twin. The non-PND group consisted of 30 cases, including 29 deliveries via cesarean section and 1 spontaneous vaginal delivery.

The characteristics of the study population are shown in Table 1. In the overall group, the mean GA at birth was 32.416 ± 2.249 weeks, and the mean birth weight of the smaller twin was 1299.200 ± 410.289 g. The GA at diagnosis in the PND group was significantly earlier than that in the non-PND group (*P* < 0.05). There was no statistical difference in the other characteristics between the PND and non-PND groups.

**Table 1:** General characteristics of the study population.

Groups	*n*	Age (years)	Height (cm)	Body weight (kg)	Nulliparous (%)	Assisted reproductive conception (%)	GA at diagnosis (weeks)
Non-PND group	30	27.93 ± 3.269	160.38 ± 4.759	56.53 ± 10.339	80.00%	6.67%	26.457 ± 3.919
PND group	19	28.42 ± 4.451	159.27 ± 3.823	53.45 ± 5.973	84.21%	5.26%	22.595 ± 3.171
*χ*^2^/z/t		−0.442	0.638	0.891	0.138	0.063	3.609
*p*-value		0.661	0.529	0.381	0.710	0.802	0.001

GA, gestational age.

### Ultrasound measurement reproducibility

The intra-observer ICC for intra-abdominal UV diameter measurement was 0.945 (95%CI: 0.798–0.979), and the inter-observer ICC was 0.879 (95%CI: 0.763–0.940), indicating excellent intra-observer repeatability and good inter-observer reproducibility.

### Comparison of ultrasound parameters between the PND group and non-PND group

We analyzed ultrasound parameters obtained at the first diagnosis of type 1 sFGR (Table 2). The UV/AC of the smaller twin in the PND group was significantly lower than that in the non-PND group (*P* < 0.05). The intertwin UV/AC discordance in the PND group were significantly higher than that in the non-PND group (*P* < 0.05). Other parameters showed no significant differences between the groups.

**Table 2:** Comparison of ultrasound parameters at the initial diagnosis of type 1 sFGR between PND group and non-PND group.

Variables	Non-PND group	PND group	t/z	*p*-value
Smaller twin UV/AC (%)	1.486 ± 0.309	1.276 ± 0.226	2.556	0.014
Intertwin UV/AC discordance (%)	0.287 (0.102–0.373)	0.543 (0.322–0.747)	−3.673	0.001
Smaller twin UA-S/D	4.503 (3.155–5.428)	4.964 (3.590–6.1510)	−1.129	0.266
Smaller twin UA-PI	1.368 ± 0.354	1.518 ± 0.376	−1.418	0.163
Smaller twin MCA-PSV	36.649 ± 11.360	33.065 ± 8.018	1.197	0.237
Smaller twin MCA-PI	1.540 ± 0.260	1.434 ± 0.300	1.311	0.196
Smaller twin CPR	1.204 ± 0.394	0.998 ± 0.311	1.925	0.060
△EFW (%)	31.033 (25.750–34.000)	36.474 (29.000–44.000)	−1.924	0.054

UV/AC, Ratio of intra-abdominal umbilical vein diameter to abdominal circumference; △EFW: intertwin estimated fetal weight values to centiles discordance; UA-S/D, umbilical artery S/D; UA-PI, umbilical artery pulsatility index; MCA-PSV, middle cerebral artery-peak systolic velocity; MCA-PI, middle cerebral artery pulsatility index; CPR, cerebroplacental ratio.

### Pearson correlation analysis of ultrasonic parameters

The intertwin UV/AC discordance was negatively correlated with the UV/AC of the smaller twin and positively correlated with △EFW. There was a negative correlation between CPR and △EFW in the smaller twin. The correlations were statistically significant (*P* < 0.05) (Table 3).

**Table 3:** Pearson correlation analysis of ultrasonic parameters at the initial diagnosis of type 1 sFGR.

Variables	Smaller twin UV/AC (%)	Intertwin UV/AC discordance (%)	Smaller twin CPR	△EFW (%)
Smaller twin UV/AC (%)	1			
Intertwin UV/AC discordance (%)	−0.522**	1		
Smaller twin CPR	0.246	−0.395**	1	
△EFW(%)	−0.040	0.338*	−0.330*	1

* *P* < 0.05.

** *P* < 0.01.

UV/AC, Ratio of intra-abdominal umbilical vein diameter to abdominal circumference; △EFW, intertwin estimated fetal weight values to centiles discordance; CPR: cerebroplacental ratio.

### Multivariate logistic regression analysis of fetal ultrasound parameters at the first diagnosis of type 1 sFGR and PND of the smaller twin

In univariate analysis, intertwin UV/AC discordance (*P* < 0.01) and GA at diagnosis (*P* < 0.01) were included in logistic regression analysis. Pearson correlation analysis showed a significant correlation between the smaller twin UV/AC and intertwin UV/AC discordance. To avoid multicollinearity and improve model stability, only intertwin UV/AC discordance was incorporated into the final multivariate logistic regression model. We also included smaller twin CPR (*P* = 0.06) and △EFW (*P* = 0.054) into logistic regression analysis, as these parameters were generally considered instructive in the clinic. There were no correlations between smaller twin CPR, △EFW, and PND (*P* > 0.05); only the Z- intertwin UV/AC discordance(*P* = 0.006) and GA at diagnosis (*P* = 0.013) was correlated with PND (Table 4).

**Table 4:** Multivariate logistic regression analysis of fetal ultrasound parameters at the first diagnosis of type 1 sFGR and PND of the smaller twin.

Variables	*β*	SE	Wald*χ*^2^ value	*P*-value	OR (95% CI)
GA at diagnosis (weeks)	−0.407	0.164	6.147	0.013	0.666 (0.482–0.918)
Z-Intertwin UV/AC discordance (%)	1.311	0.481	7.423	0.006	3.708 (1.445–9.519)
Smaller twin CPR	1.023	1.408	0.527	0.468	2.781 (0.176–43.945)
△EFW (%)	0.094	0.061	2.387	0.122	1.099 (0.975–1.238)

UV/AC, Ratio of intra-abdominal umbilical vein diameter to abdominal circumference; △EFW, intertwin estimated fetal weight values to centiles discordance; CPR, cerebroplacental ratio. Z-Intertwin UV/AC discordance, Z-score standardized value of inter-twin UV/AC discordance.

The goodness-of-fit of the multivariate logistic regression model was evaluated using the Hosmer-Lemeshow test, which indicated an adequate model fit (*χ*^2^ = 4.180, df = 8, *P* = 0.840). The Cox–Snell R² and Nagelkerke R^2^ were 0.421 and 0.572.

To further evaluate the incremental prognostic value of intertwin UV/AC discordance, nested logistic regression models were constructed: Mod A was the base model containing GA at diagnosis, smaller-twin CPR, and △EFW, while Mod B was the full model adding Z-score standardized intertwin UV/AC discordance on the basis of Mod A.

### ROC curve analysis of intertwin UV/AC discordance and GA at diagnosis for PND in type 1 sFGR

ROC curve analysis was further performed to assess the discriminatory capacity of factors for PND in type 1 sFGR smaller fetuses (Figure 2). The detailed parameters are summarized in Table 5. The intertwin UV/AC discordance achieved an AUC of 0.814 (95% CI: 0.695–0.933). At the optimal cut-off value of 0.273%, it achieved a sensitivity of 89.5% and a specificity of 53.3%, with a positive likelihood ratio of 1.920 and a negative likelihood ratio of 0.200. For GA at diagnosis, the AUC was 0.236 (95% CI: 0.103–0.369), which was less than 0.5, indicating a reverse predictive direction. Specifically, a lower GA at diagnosis was correlated with a higher risk of PND. The optimal cut-off value was 22.75 weeks, with a sensitivity of 83.3%, a specificity of 47.4%, a positive likelihood ratio of 1.584, and a negative likelihood ratio of 0.352. Collectively, intertwin UV/AC discordance showed satisfactory discriminatory capacity for stratifying PND risk within our cohort, while GA at diagnosis served as a reverse discrimination for PND risk in type 1 sFGR pregnancies.

**Figure 2:**
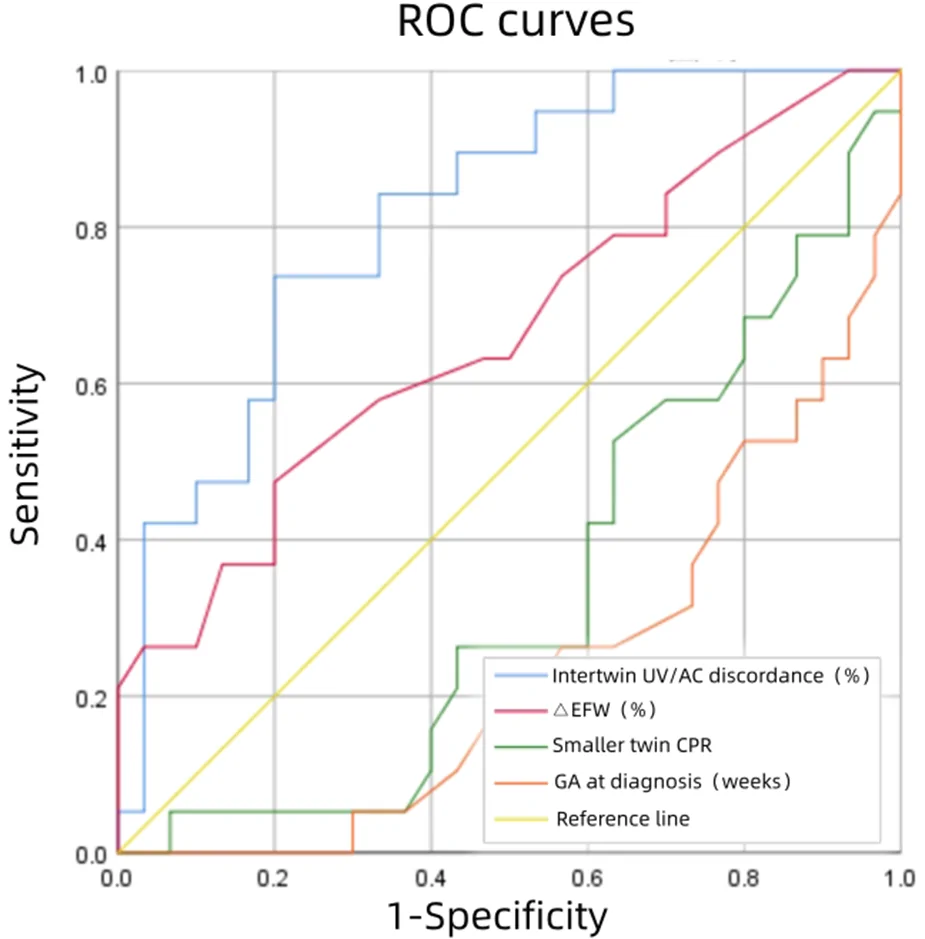
ROC curves. Receiver operating characteristic (ROC) analysis for intertwin UV/AC discordance、△EFW (%)、Smaller twin CPR and GA at diagnosis in evaluating the smaller twin with PND.

**Table 5:** The optimal cut-off point, sensitivity, specificity, positive likelihood ratio (sensitivity/(1-specificity), and negative likelihood ratio ((1-sensitivity)/specificity) of intertwin UV/AC discordance and GA at diagnosis in the evaluation of pregnancy outcome of smaller twin with type 1 sFGR.

Parameters	AUC	((95%CI)	Optimal cut-off point	Sensitivity (%)	Specificity (%)	LR+	LR−
Intertwin UV/AC discordance (%)	0.814	0.695–0.933	0.273	89.5	53.3	1.920	0.200
GA at diagnosis (weeks)	0.236	0.103–0.369	22.75	83.3	47.4	1.584	0.352

An AUC value less than 0.5 indicates that a smaller value of gestational age at diagnosis corresponds to a higher risk of perinatal death. Thus, the diagnostic criterion for the item *Gestational age at diagnosis* is reversely defined in this table. GA, *gestational age*; LR+, positive likelihood ratio; LR−, negative likelihood ratio.

Further ROC analyses were performed for the nested prediction models (Figure 3). The AUC of Mod A (base model) was 0.811 (95%CI: 0.689–0.932), and the AUC of Mod B (full model) reached 0.886 (95%CI: 0.791–0.981). Although the DeLong test indicated that the difference in AUC between the two models approached statistical significance (*P* = 0.078), continuous net reclassification improvement (NRI) and integrated discrimination improvement (IDI) demonstrated significant incremental prognostic value when intertwin UV/AC discordance was added into the base model. The continuous NRI was 0.9018 (95%CI: 0.3856-1.4179, *P* = 0.00062), and the IDI was 0.1585 (95%CI: 0.0583-0.2586, *P* = 0.00192). The categorical NRI was non-significant (0.1579, *P* = 0.226), which might be attributed to the arbitrary 0.5 probability threshold for risk stratification.

**Figure 3:**
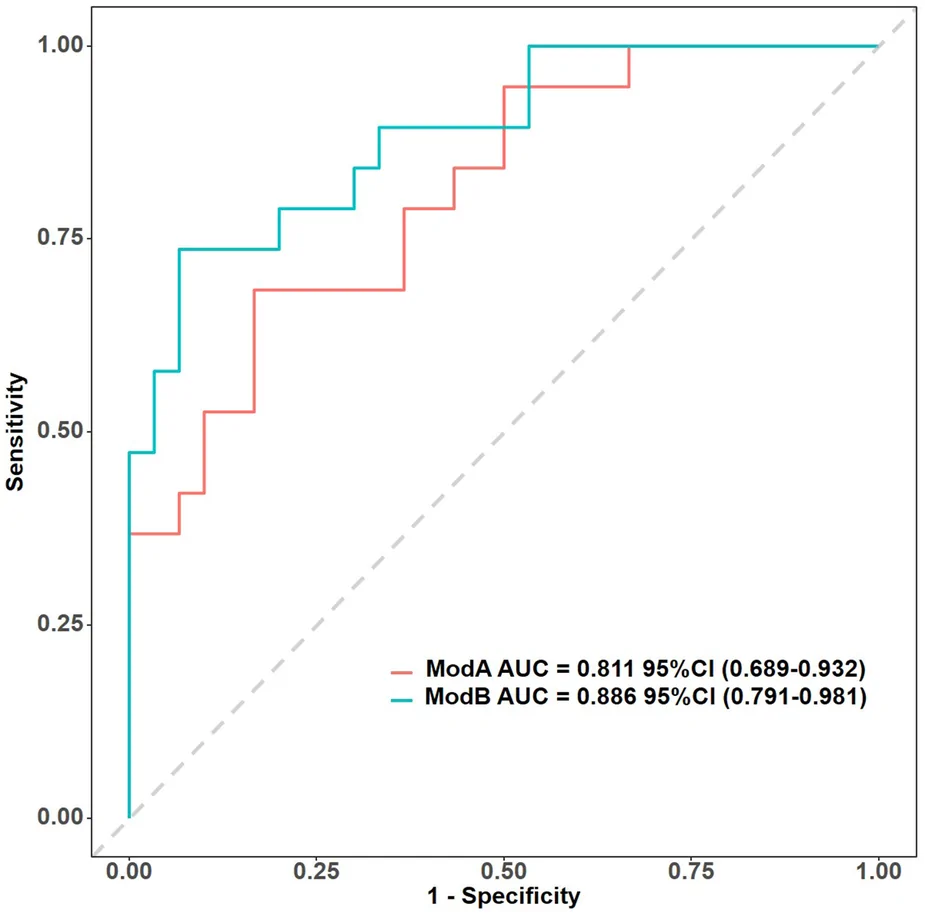
Comparison of receiver operating characteristic (ROC) curves between the base model (Mod A) and full model (Mod B). Mod A included conventional clinical and Doppler parameters, while Mod B incorporated intertwin UV/AC discordance on the basis of Mod A. AUC of Mod A = 0.811 (95%CI: 0.689–0.932), AUC of Mod B = 0.886 (95%CI: 0.791–0.981).

The apparent C-statistic (AUC) of Mod B was 0.886, with a Brier score of 0.130. Calibration curves presented acceptable consistency between model-predicted probabilities and observed outcomes (Figure 4), with an average calibration error (Eavg) of 0.037 and maximum calibration error (Emax) of 0.096; only mild risk underestimation was observed within the predicted probability range of 0.3-0.4. We conducted 500-times bootstrap internal validation to assess model optimism and potential overfitting. Decision-curve analysis indicated that Mod B provided higher net clinical benefit than both “treat-all” and “treat-none” strategies across the threshold probability of 0-0.4, while net benefit decreased substantially when threshold probability exceeded 0.4 (Figure 5).

**Figure 4:**
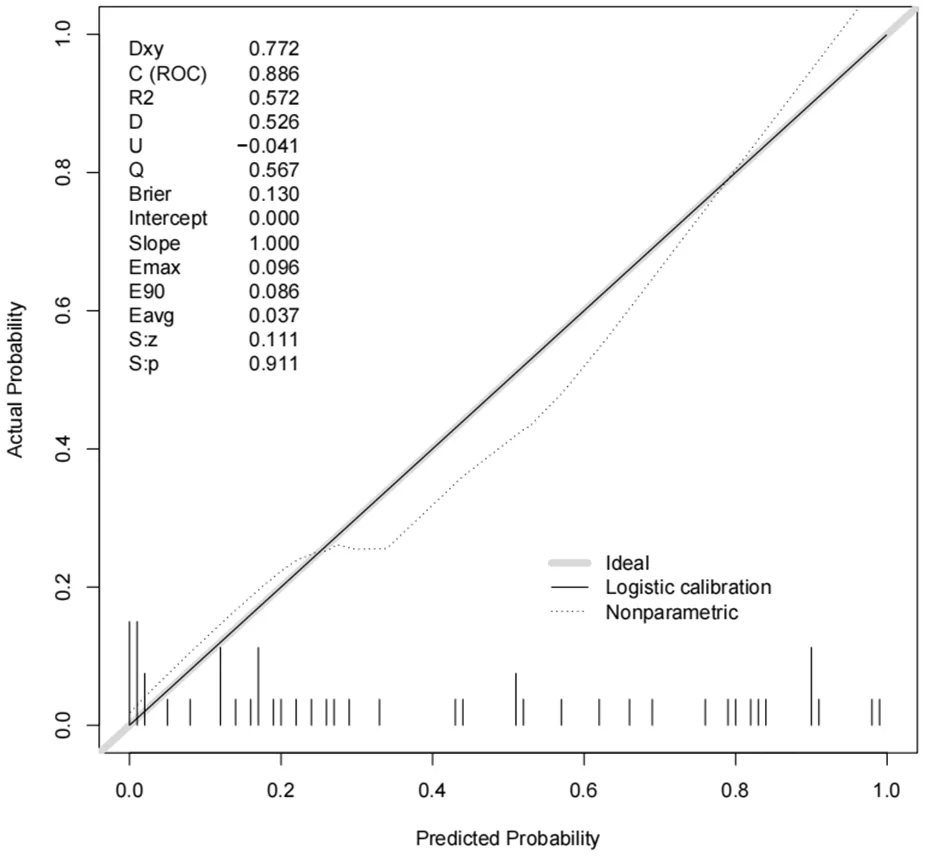
Calibration plot for the full prediction model (Mod B). The grey solid line represents ideal calibration, the black solid line denotes logistic calibration, and the dotted dashed line indicates non-parametric fit. The vertical ticks at the bottom represent the distribution of predicted probabilities for individual participants. Eavg = 0.037, Emax = 0.096, Brier score = 0.130. Acceptable overall calibration was observed, with only slight risk underestimation in the predicted probability range of 0.3-0.4.

**Figure 5:**
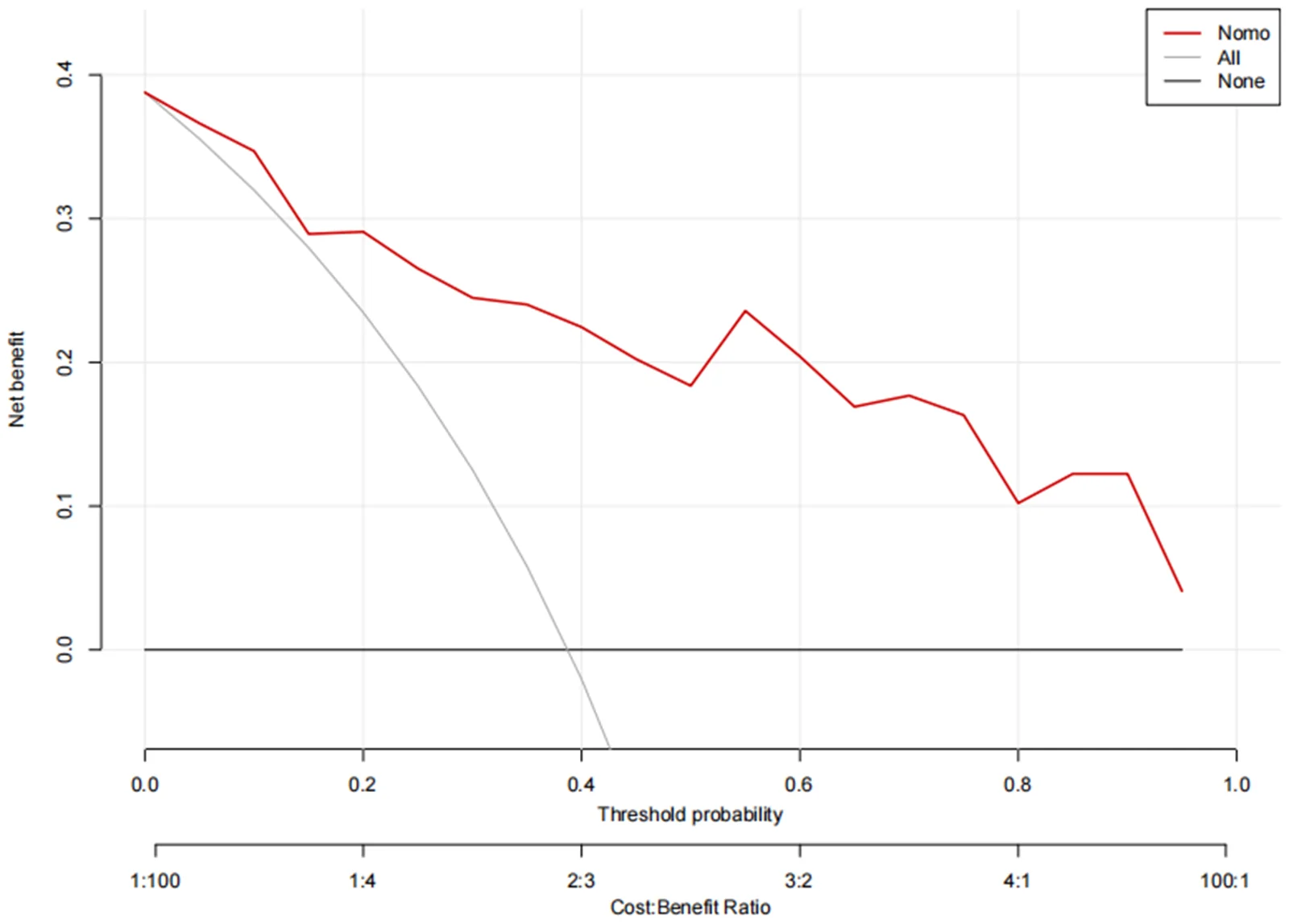
Decision-curve analysis (DCA) for the full prediction model (Mod B). The red curve represents the full prediction model; the grey oblique line represents the strategy of treating all patients; the black horizontal line represents the strategy of treating none. Net clinical benefit was observed within threshold probability 0-0.4, and net benefit decreased substantially above threshold probability of 0.4.

## Discussion

The present study systematically analyzed multiple ultrasonic parameters in MCDA twin pregnancies complicated by type 1 sFGR, aiming to identify potential prognostic markers for for PND in smaller twin. Based on univariate and multivariate logistic regression analyses, intertwin UV/AC discordance was independently associated with higher odds of PND. In addition, earlier GA at type 1 sFGR diagnosis served as a significant reverse risk factor, whereas conventional ultrasonic indicators, including umbilical artery Doppler parameters, CPR, and △EFW, exhibited limited independent predictive value in type 1 sFGR.

The occurrence and progression of sFGR are predominantly driven by unequal placental sharing and extensive inter-twin vascular anastomoses, which lead to heterogeneous oxygen and nutrient perfusion between twin fetuses (6). Prenatal ultrasound cannot accurately quantify placental distribution, while abnormal umbilical cord insertion and velamentous placenta have been well recognized as high-risk morphological features of sFGR (19, 20), although such placental abnormalities are difficult to reliably detect in late gestation. Growing evidence has indicated that early-onset and late-onset sFGR may result from distinct pathogenic mechanisms, and fetal weight discordance is not linearly correlated with placental share discrepancy, further verifying the compensatory and protective effects of inter-twin vascular anastomoses in MCDA twins (21, 22). Consistent with previous findings (21, 23–25), our study confirmed that earlier GA at diagnosis is a critical adverse prognostic factor for type 1 sFGR. Early sFGR onset indicates insufficient placental compensatory capacity and prolonged intrauterine hypoperfusion stress, which significantly increases the likelihood of adverse perinatal events. In the present cohort, conventional UA-S/D, UA-PI, and △EFW showed no prognostic significance. Previous studies have demonstrated that substantial △EFW is closely related to poor perinatal outcomes in severe sFGR cases (13, 14). The negative finding in our study is reasonably explained by the mild clinical phenotype of type 1 sFGR, which is characterized by subtle growth imbalance at diagnosis, making traditional growth and umbilical arterial parameters insensitive for early risk stratification in this specific population.

The CPR is a well-established sensitive marker reflecting cerebral hemodynamic redistribution and fetal hypoxia, with high predictive value for adverse outcomes in singleton fetal growth restriction (26). Several studies have also suggested that MCA hemodynamic discrepancies are correlated with birth weight discordance in MCDA twin pregnancies (15). However, the prognostic performance of conventional cerebral hemodynamic indices is weakened in twin sFGR, which can be attributed to the unique hemodynamic characteristics of MCDA twins. Inter-twin vascular anastomoses provide an alternative compensatory perfusion pathway, through which the larger twin can supply blood and oxygen to the hypoperfused smaller twin, stabilize cerebral circulation, and offset the typical brain-sparing response under hypoxia. This unique compensatory mechanism effectively diminishes the predictive efficacy of conventional cerebral hemodynamic parameters for PND in type 1 sFGR.

UV circulation directly reflects placental oxygen and nutrient transport function, serving as a more reliable indicator of overall fetal nutritional supply compared with arterial Doppler parameters (27, 28). Different from ductus venosus indices, which are highly susceptible to fetal posture and gestational age, technically difficult to measure, and occasionally unobtainable due to congenital absence or premature closure (27, 29–31), intra-abdominal UV diameter measurement is stable, convenient, and highly feasible for routine prenatal surveillance. Accumulated studies on singleton FGR have confirmed that impaired UV perfusion and abnormal venous shunting are closely associated with fetal liver hypoperfusion and adverse perinatal outcomes (32–34). Recent twin-related studies have further demonstrated that UV structural and functional abnormalities are predictive of fetal acidemia and intrauterine demise, and early inter-twin UV discrepancy is linked to elevated fetal mortality risk in MCDA twin pregnancies (35, 36). In line with previous perspectives, inter-twin hemodynamic discrepancy in MCDA twins primarily reflects unequal placental sharing rather than birth weight imbalance (37). In this study, intertwin UV/AC discordance eliminated individual fetal differences and reflected the true degree of placental perfusion inequality between twins in type 1 sFGR. Combined with the favorable discriminative performance observed in ROC analysis, intertwin UV/AC discordance exhibits potential and stable prognostic value for early risk stratification in type 1 sFGR.

Nevertheless, the cohort-derived cutoff for intertwin UV/AC discordance had a low specificity of 53.3%, which may produce false-positive findings. This threshold should not guide clinical decisions alone and requires integration with other clinical and sonographic data. There is an inherent sensitivity-specificity trade-off; we prioritized higher sensitivity to minimize missed high-risk cases, with a corresponding reduction in specificity.

When added to the conventional clinical model, intertwin UV/AC discordance improved risk-stratification performance. While the AUC difference was only marginally significant, reclassification analyses suggested that this venous parameter provided incremental prognostic value beyond routine clinical and Doppler indices.

Clinically, the optimal surveillance strategy and delivery timing for type 1 sFGR remain controversial. Expectant management prolongs fetal exposure to progressive placental dysfunction and intrauterine hypoperfusion, while preterm delivery inevitably increases the risks of neonatal prematurity-related complications. Balancing these clinical trade-offs remains a major challenge in the management of type 1 sFGR. Combined assessment of intertwin UV/AC discordance and GA at diagnosis may serve as a novel auxiliary strategy for early risk stratification. It enables identification of high-risk type 1 sFGR pregnancies to guide individualized monitoring and clinical intervention, which may help optimize perinatal management in high-risk MCDA twin pregnancies.

This study has several limitations. As a single-center retrospective study, the sample size was relatively limited due to the inherent rarity of type 1 sFGR in MCDA twin pregnancies, and potential selection bias cannot be completely excluded. The full prediction model yielded an events-per-variable (EPV) of approximately 4.75, which raises the potential risk of model overfitting. Although bootstrap internal validation was conducted to assess optimistic bias of the model, external verification in independent cohorts is still warranted. Moreover, although both intra-observer and inter-observer reproducibility of intra-abdominal UV diameter measurement were confirmed to be good, this study lacked external validation of the cut-off value for intertwin UV/AC discordance. Despite these limitations, the present study innovatively explored the prognostic value of intra-abdominal UV-derived parameters in type 1 sFGR. We demonstrated that intertwin UV/AC discordance is independently associated with PND in type 1 sFGR smaller twin. This finding enriches the existing ultrasonic prognostic framework for sFGR, provides novel insights into the underlying pathophysiological mechanisms of twin growth restriction, and exhibits its potential value for antenatal risk assessment in clinical practice.

## Conclusions

This study explored ultrasound-derived factors associated with PND in smaller twin with type 1 sFGR, focusing on the novel intertwin UV/AC discordance. The results demonstrated that higher intertwin UV/AC discordance is independently associated with increased PND risk, while earlier diagnostic GA constitutes an important inverse risk factor. The combination of intertwin UV/AC discordance and GA at diagnosis may facilitate risk-stratified antenatal assessment and support individualized management for type 1 sFGR. Such a combined assessment has the potential to optimize surveillance strategies for high-risk MCDA twin pregnancies.

## Data Availability

The original contributions presented in the study are included in the article/Supplementary Material, further inquiries can be directed to the corresponding author/s.

## References

[1] Khalil A Beune I Hecher K Wynia K Ganzevoort W Reed K. Consensus definition and essential reporting parameters of selective fetal growth restriction in twin pregnancy: a delphi procedure. Ultrasound Obstet Gynecol. (2019) 53(1):47–54. 10.1002/uog.19013 29363848

[2] Sebire NJ Snijders RJ Hughes K Sepulveda W Nicolaides KH. The hidden mortality of monochorionic twin pregnancies. Br J Obstet Gynaecol. (1997) 104(10):1203–7. 10.1111/j.1471-0528.1997.tb10948.x 9333002

[3] Inklaar MJ Van Klink JM Stolk TT Van Zwet EW Oepkes D Lopriore E. Cerebral injury in monochorionic twins with selective intrauterine growth restriction: a systematic review. Prenat Diagn. (2014) 34(3):205–13. 10.1002/pd.4298 24338685

[4] Hack KEA Derks JB Elias SG Franx A Roos EJ Voerman SK. Increased perinatal mortality and morbidity in monochorionic versus dichorionic twin pregnancies: clinical implications of a large Dutch cohort study. Obstet Anesth Digest. (2008) 28(3):156–7. 10.1097/01.aoa.0000326398.59896.a6 17999692

[5] Southwest Thames Obstetric Research Collaborative (STORK). Prospective risk of late stillbirth in monochorionic twins: a regional cohort study. Ultrasound Obstet Gynecol. (2012) 39(5):500–4. 10.1002/uog.11110 22302586

[6] Lewi L Cannie M Blickstein I Jani J Huber A Hecher K. Placental sharing, birthweight discordance, and vascular anastomoses in monochorionic diamniotic twin placentas. Am J Obstet Gynecol. (2007) 197(6):587.e1–e8. 10.1016/j.ajog.2007.05.009 18060944

[7] Khalil A Rodgers M Baschat A Bhide A Gratacos E Hecher K. ISUOG practice guidelines: role of ultrasound in twin pregnancy. Ultrasound Obstet Gynecol. (2016) 47(2):247–63. 10.1002/uog.15821 26577371

[8] Breathnach FM McAuliffe FM Geary M Daly S Higgins JR Dornan J. Definition of intertwin birth weight discordance. Obstet Gynecol. (2011) 118(1):94–103. 10.1097/AOG.0b013e31821fd208 21691168

[9] D'Antonio F Khalil A Morlando M Thilaganathan B. Accuracy of predicting fetal loss in twin pregnancies using gestational age-dependent weight discordance cut-offs: analysis of the STORK multiple pregnancy cohort. Fetal Diagn Ther. (2015) 38(1):22–8. 10.1159/000369326 25660975

[10] Mulcahy C McAuliffe FM Breathnach F Geary M Daly S Higgins J. Umbilical and fetal middle cerebral artery Doppler reference ranges in a twin population followed longitudinally from 24 to 38 weeks’ gestation. Ultrasound Obstet Gynecol. (2014) 44(4):461–7. 10.1002/uog.13302 24407772

[11] Qiu T Cheng W Chen Y Guo Y Shen H Xu L. Clinical outcome of monochorionic diamniotic twins with intrauterine growth restriction. Am J Perinat. (2024) 41(10):1321–8. 10.1055/s-0042-1754394 PMC11223781 38154467

[12] Buca D Pagani G Rizzo G Familiari A Flacco ME Manzoli L. Outcome of monochorionic twin pregnancy with selective intrauterine growth restriction according to umbilical artery Doppler flow pattern of smaller twin: systematic review and meta-analysis. Ultrasound Obstet Gynecol. (2017) 50(5):559–68. 10.1002/uog.17362 27859836

[13] D'Antonio F Odibo AO Prefumo F Khalil A Buca D Flacco ME. Weight discordance and perinatal mortality in twin pregnancy: systematic review and meta-analysis. Ultrasound Obstet Gynecol. (2018) 52(1):11–23. 10.1002/uog.18966 29155475

[14] D'Antonio F Khalil A Dias T Thilaganathan B. Weight discordance and perinatal mortality in twins: analysis of the southwest thames obstetric research collaborative (STORK) multiple pregnancy cohort. Ultrasound Obstet Gynecol. (2013) 41(6):643–8. 10.1002/uog.12412 23355123

[15] Stagnati V Pagani G Fichera A Prefumo F. Intertwin discrepancy in middle cerebral artery peak systolic velocity and third-trimester fetal growth restriction in monochorionic-diamniotic twin pregnancy. Ultrasound Obstet Gynecol. (2016) 48(1):66–71. 10.1002/uog.14944 26173065

[16] Gratacós E Lewi L Muñoz B Acosta-Rojas R Hernandez-Andrade E Martinez JM. A classification system for selective intrauterine growth restriction in monochorionic pregnancies according to umbilical artery Doppler flow in the smaller twin. Ultrasound Obstet Gynecol. (2007) 30(1):28–34. 10.1002/uog.4046 17542039

[17] Şeker E Çimen Y Süt H Ümit C Koçar M Yıldız GA. Predicting fetal distress in growth-restricted fetuses: the role of umbilical vein diameter. J Obstet Gynaecol Res. (2025) 51(9):e70082. 10.1111/jog.70082 40962300

[18] Parsaei M Zamanpour Z Ghasemipour Afshar Z Noorafrooz M Dashtkoohi M Taghva Nakhjiri M. Umbilical vein Doppler evaluation in small-for-gestational-age fetuses with normal umbilical artery Doppler findings: a prospective cohort study. J Ultrasound Med. (2025) 44(12):2257–69. 10.1002/jum.70007 40781982

[19] Costa-Castro T Zhao DP Lipa M Haak MC Oepkes D Severo M. Velamentous cord insertion in dichorionic and monochorionic twin pregnancies—does it make a difference? Placenta. (2016) 42:87–92. 10.1016/j.placenta.2016.04.007 27238718

[20] Kalafat E Thilaganathan B Papageorghiou A Bhide A Khalil A. Significance of placental cord insertion site in twin pregnancy. Ultrasound Obstet Gynecol. (2018) 52(3):378–84. 10.1002/uog.18914 28976606

[21] Lewi L Gucciardo L Huber A Jani J Van Mieghem T Doné E. Clinical outcome and placental characteristics of monochorionic diamniotic twin pairs with early- and late-onset discordant growth. Am J Obstet Gynecol. (2008) 199(5):511.e1–e7. 10.1016/j.ajog.2008.04.022 18539257

[22] Noll ATR Lof FC Groene SG Haak MC Lopriore E Russo FM. Artery-to-vein anastomoses in unequally divided placentas and their association with birthweight discordance. Placenta. (2024) 146:58–63. 10.1016/j.placenta.2023.12.023 38181521

[23] Monaghan C Kalafat E Binder J Thilaganathan B Khalil A. Prediction of adverse pregnancy outcome in monochorionic diamniotic twin pregnancy complicated by selective fetal growth restriction. Ultrasound Obstet Gynecol. (2019) 53(2):200–7. 10.1002/uog.19078 29704280

[24] Curado J Sileo F Bhide A Thilaganathan B Khalil A. Early- and late-onset selective fetal growth restriction in monochorionic diamniotic twin pregnancy: natural history and diagnostic criteria. Ultrasound Obstet Gynecol. (2020) 55(5):661–6. 10.1002/uog.20849 31432560

[25] Batsry L Matatyahu N Avnet H Weisz B Lipitz S Mazaki-Tovi S. Perinatal outcome of x diamniotic twin pregnancy complicated by selective intrauterine growth restriction according to umbilical artery Doppler flow pattern: single-center study using strict fetal surveillance protocol. Ultrasound Obstet Gynecol. (2021) 57(5):748–55. 10.1002/uog.22128 32573847

[26] Rizzo G Mappa I Bitsadze V Słodki M Khizroeva J Makatsariya A. Role of Doppler ultrasound at time of diagnosis of late-onset fetal growth restriction in predicting adverse perinatal outcome: prospective cohort study. Ultrasound Obstet Gynecol. (2020) 55(6):793–8. 10.1002/uog.20406 31343783

[27] Parra-Saavedra M Crovetto F Triunfo S Savchev S Parra G Sanz M. Added value of umbilical vein flow as a predictor of perinatal outcome in term small-for-gestational-age fetuses. Ultrasound Obstet Gynecol. (2013) 42(2):189–95. 10.1002/uog.12380 23288780

[28] Cox LA Schlabritz-Loutsevitch N Hubbard GB Nijland MJ McDonald TJ Nathanielsz PW. Gene expression profile differences in left and right liver lobes from mid-gestation fetal baboons: a cautionary tale. J Physiol. (2006) 572(Pt 1):59–66. 10.1113/jphysiol.2006.105726 16484296 PMC1779658

[29] Seo N Kurihara Y Suekane T Yokoi N Nakagawa K Tahara M. Altered transmission of cardiac cycles to ductus venosus blood flow in fetal growth restriction: why ductus venosus reflects fetal circulatory changes more precisely. Diagnostics (Basel). (2022) 12(6):1393. 10.3390/diagnostics12061393 35741203 PMC9221754

[30] Ferrazzi E Rigano S Bozzo M Bellotti M Giovannini N Galan H. Umbilical vein blood flow in growth-restricted fetuses. Ultrasound Obstet Gynecol. (2000) 16(5):432–8. 10.1046/j.1469-0705.2000.00208.x 11169327

[31] Farsetti D Pometti F Tiralongo GM Lo Presti D Pisani I Gagliardi G. Distinction between SGA and FGR by means of fetal umbilical vein flow and maternal hemodynamics. J Matern Fetal Neonatal Med. (2022) 35(25):6593–9. 10.1080/14767058.2021.1918091 33938366

[32] Bellotti M Pennati G D Gasperi C Bozzo M Battaglia FC Ferrazzi E. Simultaneous measurements of umbilical venous, fetal hepatic, and ductus venosus blood flow in growth-restricted human fetuses. Am J Obstet Gynecol. (2004) 190(5):1347–58. 10.1016/j.ajog.2003.11.018 15167841

[33] Valensise H Farsetti D Pometti F Vasapollo B Novelli GP Lees C. The cardiac-fetal-placental unit: fetal umbilical vein flow rate is linked to the maternal cardiac profile in fetal growth restriction. Am J Obstet Gynecol. (2023) 228(2):222.e1–222.e12. 10.1016/j.ajog.2022.08.004 35944606

[34] Triunfo S Crispi F Gratacos E Figueras F. Prediction of delivery of small-for-gestational-age neonates and adverse perinatal outcome by fetoplacental Doppler at 37 weeks’ gestation. Ultrasound Obstet Gynecol. (2017) 49(3):364–71. 10.1002/uog.15979 27241184

[35] Liao TB Nomura RM Liao AW Francisco RP Zugaib M. Fetal venous circulation in monochorionic twin pregnancies with placental insufficiency: prediction of acidemia at birth or intrauterine fetal death. Ultrasound Obstet Gynecol. (2014) 43(4):426–31. 10.1002/uog.12549 23828752

[36] Couck I Van Der Merwe J Russo F Richter J Aertsen M Cauwberghs B. Umbilical venous diameter and flow in monochorionic diamniotic twin pregnancy: association with placental sharing and fetal demise. Ultrasound Obstet Gynecol. (2022) 60(4):514–22. 10.1002/uog.24903 35316571

[37] Chang YL Chang SD Chao AS Wang CN Wang TH Cheng PJ. The relationships of umbilical venous volume flow, birthweight and placental share in monochorionic twin pregnancies with and without selective intrauterine growth restriction. Twin Res Hum Genet. (2011) 14(2):192–7. 10.1375/twin.14.2.192 21425903

